# Psychometric study of the Hospital Anxiety and Depression Scale with health professionals

**DOI:** 10.11606/s1518-8787.2025059006420

**Published:** 2025-06-11

**Authors:** Priscila Pereira da Silva Lopes, Rafaela Schaefer, Juliana Nichterwitz Scherer

**Affiliations:** I Universidade do Vale do Rio dos Sinos Programa de Pós-Graduação em Saúde Coletiva São Leopoldo RS Brasil Universidade do Vale do Rio dos Sinos. Programa de Pós-Graduação em Saúde Coletiva. São Leopoldo, RS, Brasil

**Keywords:** Validation Study, Psychometrics, Anxiety, Depression, Health Care Workers, Primary Health Care

## Abstract

**OBJECTIVE:**

To assess the validity of the internal structure and reliability of the Brazilian version of the Hospital Anxiety and Depression Scale (HADS) for screening depressive and anxious symptoms in primary care health professionals.

**METHODS:**

A psychometric study carried out with health professionals from primary health care services in a municipality in Rio Grande do Sul, Brazil, in 2023. The quality of the HADS instrument was assessed by internal structural validity, using Exploratory Factor Analysis (EFA), Confirmatory Factor Analysis (CFA), and internal consistency, using Cronbach’s Alpha and McDonald’s Omega coefficients

**RESULTS:**

217 health professionals took part in the study, of both sexes and from different professional categories, such as doctors, nurses, nursing technicians and assistants, dentists and oral health assistants, and community health workers. The EFA showed the two-factor model, anxiety and depression, as originally proposed. The CFA confirmed that the two-dimensional model was a good fit for the sample investigated. The Alpha and Omega values were 0.833 and 0.838 for anxiety and 0.763 and 0.766 for depression, respectively. Both indicated acceptable reliability.

**CONCLUSIONS:**

The HADS is a valid and reliable instrument for screening symptoms of anxiety and depression in primary care professionals in Rio Grande do Sul, Brazil.

## INTRODUCTION

Anxiety and depression are common symptoms in the general population^1^and in the working population^2^, including health professionals^3^. In a cross-sectional study carried out in 2017 in primary health care (PHC) in a municipality in São Paulo, Brazil, anxiety and depression were present in 45.3% and 41.0% of professionals, respectively^4^.

Anxiety and depressive symptoms directly and indirectly cause individual and social costs for health professionals and services, as they are related to: professional well-being^5^, professional turnover^6^, presenteeism, absenteeism^7^, and the quality of care provided^5^. For this reason, it is important to screen for these symptoms to identify health and disease conditions and to guide intervention strategies for promotion and prevention^5^.

Screening for symptoms of anxiety and depression is usually done using measuring instruments such as scales and questionnaires. Among the various instruments available in the literature is the Hospital Anxiety and Depression Scale (HADS)^8,9^. The HADS was originally developed in the United States of America (USA) for use with non-psychiatric patients in a hospital environment^10^and was translated and validated for Brazil in 1995 by Botega et al.^11^. It is a self-administered scale that is quick to complete, easy to understand and does not require prior training. These characteristics make it one of the most widely used instruments for screening symptoms of anxiety and depression^12^in different populations, including health professionals^13,14^. However, many of these studies lack an evaluation of the psychometric properties of the HADS for the population and context investigated.

Psychometrics can be used to assess the quality of measuring instruments, with validity and reliability being their main characteristics^15^. Validity is the property that an instrument measures exactly what it sets out to. Among the types of validity is internal structural validity, which refers to “the degree to which evidence and theory support interpretations of test results”^16^. Reliability is the ability of an instrument to reproduce accurate results. Both properties provide necessary evidence about the quality of the instruments and their results, whether they are valid and reliable for the sample being researched^15^.

Considering that no psychometric study of the HADS applied to primary care health professionals in Brazil has been identified in the literature, this research aimed to determine the validity of the internal structure and reliability of the Brazilian version of the HADS for screening anxiety and depression in primary care health professionals in a municipality in the metropolitan region of Porto Alegre, in Rio Grande do Sul.

## METHODS

### Design

This is a psychometric study of the HADS instrument. This process included validating the internal structure and analyzing the reliability of the scale. This type of study contributes to the rigor of scientific research by evaluating the data collection instruments themselves^17^. Psychometrics is based on measurement by theory, i.e. it uses numbers and a theoretical basis to ensure the validity and reliability of the results^15^.

### Main Study

This research is linked to the cross-sectional study: “Organizational Context of Work in Health and Workers’ Health: an analysis of the ethical climate and associated factors”, developed by the Postgraduate Program in Collective Health at the University of Vale do Rio dos Sinos (Unisinos) and funded by the Fundação de Amparo à Pesquisa do Estado do Rio Grande do Sul (Fapergs) through call 10/2021 (ARD/ARC).

### Participants and Study Site

The study population was made up of health professionals working in the primary care services of a municipality in the metropolitan region of Porto Alegre, located in the state of Rio Grande do Sul, including doctors, nurses, nursing technicians and assistants, dentists, oral health assistants, community health workers, physiotherapists, and psychologists.

### Procedures

The sample was selected using the convenience method. Participants were recruited after a brief presentation of the study to the teams of all the basic health units (UBS) in the municipality under investigation, by prior appointment, with a subsequent formal invitation to take part in the research. The final sample consisted of 217 health professionals. Participants included health professionals who worked in care and excluded those in exclusively administrative positions and residents. Data collection took place in person in April and May 2023, using printed questionnaires completed by the participants themselves, addressing sociodemographic questions, the HADS and other questions related to the other outcomes present in the larger project

### Instrument

The HADS was developed by Zigmond and Snaith in 1983^10^. It is a quick, self-administered screening instrument in which the participant responds based on how they have felt over the last week. The aim of the scale is to investigate symptoms of anxiety and depression, without the intention of determining a clinical diagnosis. The HADS is made up of two subscales, the HADS-A (anxiety subscale - odd items) and the HADS-D (depression subscale - even items) using a Likert scale from 0 to 3. To interpret the results, the scores for each subscale were added up, ranging from 0 to 21 points, with 8 being the cut-off point^10,11^. This study used the Brazilian version of the HADS, translated and adapted by Botega et al.^11^.

### Statistical Analysis

Statistical analyses were carried out using the software JASP^®^ version 0.18.1 and JAMOVI^®^ version 2.4.1. Descriptive analyses of the sample were applied, estimating absolute and relative frequencies, Exploratory Factor Analysis (EFA) and Confirmatory Factor Analysis (CFA) to assess the validity of the internal structure and Cronbach’s Alpha (α) and McDonald’s Omega (ω) coefficients to assess reliability.

The EFA used the Principal Axis Factorization extraction method in combination with Varimax rotation. The following criteria were used for the EFA: Kaiser-Meyer-Olkin Index (KMO) p> 0.05, Bartlett’s test of sphericity p< 0.05 and factor loadings equal to or greater than 0.50^21^.

In the CFA, the parameters were estimated using the robust DWLS (Diagonally Weighted Least Squares) method. The CFA indices recommended in the literature and used in this study were: CFI (Comparative Fit Index), TLI (Tucker-Lewis Index), RMSEA (Root-Mean-Square Error of Approximation) and SRMR (Standardized Root Mean Square Residual)^18,19^. The acceptable and expected reference values to consider a good model fit were: CFI and TLI ≥ 0.90 acceptable or ≥ 0.95 expected, RMSEA ≤ 0.06 acceptable or ≤ 0.05 expected, SRMR ≤ 0.08 acceptable or ≤ 0.05 expected^18-20^. In addition to these indices, the chi-squared ratio (χ^2^) was used in relation to the degrees of freedom (χ^2^/gl), where values of 1 to 3 indicate adequate adjustment^21,22^.

The reliability of the scale was estimated using Cronbach’s Alpha and McDonald’s Omega coefficients. For both coefficients, an adequate value was considered to be equal to or greater than 0.70^23,24^, with 0.70 being acceptable, 0.80 very acceptable and 0.90 ideal^25^. Values above 0.90 suggest item redundancy^23,26^

### Ethical Aspects

This study was approved by the Unisinos Research Ethics Committee (No. 5.456.590) and by the Municipal Center for Collective Health Education (Numesc). The information collected was kept confidential, without specifying the municipality’s UBS and without identifying the individuals. All participants were informed of the study’s objectives and signed an informed consent form.

## RESULTS

### Characteristics of the Participants

The sociodemographic and professional characteristics of the 217 participants are shown in Table 1. The median age was 40 years, with a predominance of female health professionals (83.4%), white skin color (74.1%), married (53.5%), belonging to the nursing professional category of technicians and assistants (31.3%), and with technical schooling (36.2%). The average HADS-A score was 6.54 (SD± 4.1) and the average HADS-D score was 5.20 (SD± 3.34) for the entire sample.

**Table 1:** Sociodemographic characteristics of participants (n = 217)

Features	n	%	HADS-A^a^	HADS-D^a^
Age (Median and [IQ])	213	40.0 [33.0; 50.0]	-	-
Sex				
Female	181	83.4	6.71 (4.16)	5.31 (3.34)
Male	36	16.6	5.67 (3.75)	4.66 (3.33)
Color				
White	160	74.1	6.37 (4.00)	5.13 (3.32)
Black	27	12.5	6.59 (4.24)	4.78 (2.97)
Brown	26	12.0	7.46 (4.68)	5.88 (3.92)
Yellow and indigenous	3	1.4	7.33 (4.93)	7.00 (3.46)
Marital status				
Single	100	46.5	6.68 (3.92)	5.29 (3.20)
Married	115	53.5	6.47 (4.29)	5.13 (3.50)
Profession				
Doctor	34	15.7	6.00 (4.22)	4.71 (3.31)
Nurse	29	13.4	6.34 (3.90)	5.07 (2.79)
Nursing technician and assistant	68	31.3	6.29 (4.10)	4.51 (2.90)
Community health agent	49	22.6	6.98 (4.12)	6.60 (3.76)
Dentist and oral health assistant	33	15.2	6.81 (4.45)	5.03 (3.69)
Other (physiotherapist and psychologist)	4	1.8	9.00 (1.41)	6.50 (2.08)
Education				
Elementary	3	1.4	6.33 (5.13)	6.00 (5.66)
High scool	37	17.4	6.64 (4.01)	5.97 (4.13)
Technical	77	36.2	6.66 (4.39)	4.87 (3.13)
Graduation	35	16.4	6.20 (3.41)	4.86 (2.86)
Postgraduation	61	28.6	6.60 (4.24)	5.38 (3.29)

IQ: interquartile range; HADS-A: anxiety subscale; HADS-D: depression subscale

^a^ Mean (standard deviation).

### Validity of the Internal Structure of the HADS

The EFA assumptions indicated an appropriate factor matrix: KMO: 0.91 and Bartlett’s test of sphericity < 0.001. The factor loadings for each HADS item ranged from 0.53 to 0.72 for the anxiety factor and from 0.44 to 0.63 for the depression factor. Despite some borderline values, none indicated a low contribution to the corresponding subscale (Table 2).

**Table 2:** Factor loading of the HADS items with primary care health professionals.

HADS-Item	Factor Load
Anxiety subscale	
1. Nervous or tense	0.60
3. Fear of something horrible	0.72
5. Full of worry	0.72
7. Peaceful and relaxed	0.53
9. Fear and tightness in the stomach	0.70
11. Restless	0.58
13. Panic	0.67
Depression subscale	
2. Likes the same things	0.44
4. Laughter and fun	0.57
6. Feeling cheerful	0.63
8. Slowness	0.54
10. Taking care of your appearance	0.57
12. Hope for the future	0.62
14. Pleasure with books, TV, or radio	0.53

HADS: Hospital Anxiety and Depression Scale

The CFA of the total HADS and by subscales showed a good model fit for the sample under study, as shown in Table 3.

**Table 3:** Confirmatory factor analysis of the HADS.

Contents	Expected value	Acceptable value	HADS-total	Conclusion HADS-total	HADS-A	Conclusion HADS-A	HADS-D	Conclusion HADS-D
χ^2^ (gl)	-	-	129 (76)	-	18.500 (14)	-	23,6 (14)	-
p value χ^2^	> 0.05	-	< 0.001	Poor fit	0.185	Good fit	0.051	Good fit
χ^2^/gl	-	1-3	1.697	Suitable	1.320	Suitable	1.68	Suitable
CFI	≥ 0.95	0.90	0.940	Acceptable	0.990	Expected	0.963	Expected
TLI	≥ 0.95	0.90	0.929	Acceptable	0.985	Expected	0.945	Acceptable
RMSEA	≤ 0.50	0.60	0.056	Acceptable	0.038	Expected	0.056	Acceptable
90%CI RMSEA	-	-	0.039-0.073	-	0.000-0.080	-	0.000-0.094	-
SRMR	≤ 0.50	0.80	0.050	Expected	0.028	Expected	0.038	Expected

χ^2^: chi-square test; gl: degrees of freedom; CFI: comparative fit index; TLI: Tucker Lewis index; RMSEA: root mean square error of approximation; CI: confidence interval; SRMR: standardized root mean square residual

Ans: anxiety; Dep: depression; HADS1: Hospital Anxiety and Depression Scale item number 1

The Figure presents the structural model of the Brazilian version of the HADS with primary care health professionals and shows that both subscales (anxiety and depression) are positively correlated with each other.

**Figure:**
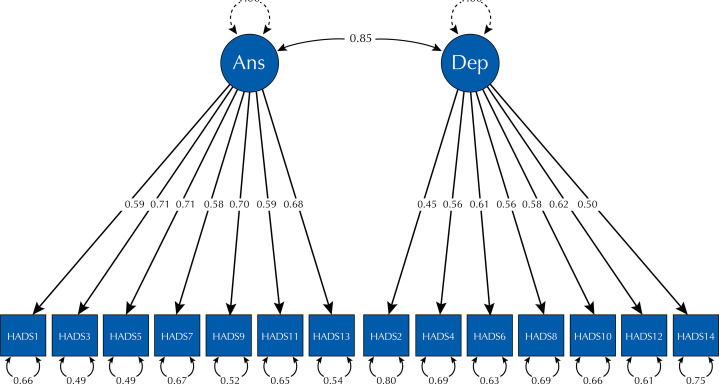
Factor diagram of the HADS for the sample under study.

### HADS Reliability

The alpha coefficient found was 0.879 for HADS-total, 0.833 for HADS-A, and 0.763 for HADS-D. In addition, the omega values were 0.881 for HADS-total, 0.838 for HADS-A, and 0.766 for HADS-D. It can be seen that the values show acceptable and very acceptable internal consistency (0.70-0.80) with no redundancy in the scale, since no value was higher than 0.90.

The change in the value of internal consistency, assessed by the alpha and omega coefficients, was tested by removing items from the scale to assess whether any item was out of sync with the scale and/or its factor, analyzing whether the scale/sub-scale would be better without it. The results show that all the items are necessary for the composition of the scale. Furthermore, the correlation between the item and the total score of the scale, presented as total-item correlation, shows an adequate correlation, except for items 2, 4, and 14 (Table 4).

**Table 4:** HADS reliability characteristics.

Item	Average (SD)	Correlation item-total	If the item is deleted	
			Cronbach’s α	McDonald’s ω
HADS-1	1.23 (0.76)	0.528	0.872	0.874
HADS-2	0.80 (0.67)	0.401	0.878	0.879
HADS-3	0.89 (0.95)	0.626	0.867	0.869
HADS-4	0.32 (0.61)	0.485	0.874	0.875
HADS-5	1.44 (0.90)	0.637	0.867	0.868
HADS-6	0.89 (0.72)	0.535	0.872	0.873
HADS-7	1.03 (0.84)	0.572	0.870	0.871
HADS-8	1.15 (0.81)	0.517	0.873	0.874
HADS-9	0.71 (0.72)	0.608	0.869	0.870
HADS-10	0.72 (0.80)	0.523	0.873	0.874
HADS-11	0.77 (0.89)	0.554	0.871	0.872
HADS-12	0.77 (0.83)	0.593	0.869	0.870
HADS-13	0.44 (0.67)	0.615	0.869	0.869
HADS-14	0.56 (0.73)	0.473	0.875	0.876

SD: standard deviation

## DISCUSSION

This study evaluated the psychometric properties of the HADS, internal structural validity and reliability, in a sample of PHC health professionals from a municipality in Rio Grande do Sul, Brazil. The results of the EFA indicated that the factor matrix of the HADS is appropriate for this population. The two-factor structure - anxiety and depression - presented a satisfactory fit in the CFA and showed acceptable reliability for the total scale and subscales by Cronbach’s Alpha and McDonald’s Omega. Furthermore, all the items were correlated with their respective factors.

A very important debate about HADS is its dimensionality. Some authors support the scale’s one-dimensional structure believing that it does not clearly differentiate the anxiety and depression constructs and thus propose that it be used as a general one-dimensional measure of emotional distress^27^. Other authors point out that a two-factor model fits the HADS better than a one-factor or three-factor model^28^. Gough and Hudon^29^explored two- and three-factor models for an Australian sample of caregivers of cancer patients and found that the best model was two-dimensional. The two-factor structure has been found in most validity studies, even with different samples, showing good fit indices and providing support for the original two-dimensional structure of the HADS^27,28,30^, corroborating the findings of our study.

The failure to differentiate the constructs does not invalidate the results of the HADS^28^, however, it is recommended that it be used as a separate measure for anxiety and depression^12^. Similar comments were made in a study with people suffering from dementia, which showed that the HADS was suitable for use in its original format, reinforcing the recommendation that the anxiety and depression factors should be measured and analyzed separately^31^.

To date, there has only been one study similar to this one, carried out with a cross-sectional sample of health professionals. Despite presenting similar indices and values and using the same base reference for the reference values in the CFA, the study was not carried out in the context of PHC and had a sample of 803 health professionals from Bangladesh^14^.

In this study, the CFA indices showed a good model fit, confirming the theoretical structure of the HADS. Analysis of the factor loadings revealed that the HADS items were clearly grouped into their respective anxiety and depression factors, corroborating the two-dimensional structure proposed in Zigmond and Snaith’s original model^10^. Only item 2, belonging to the depression factor, had a factor weight of less than 0.50 (0.44).

The internal consistency indicators (α) of the HADS were satisfactory when compared to the values recommended in the literature^23-25^and previous studies^13,14^. However, no study was found that applied McDonald’s ω with health professionals to assess reliability, which makes it difficult to compare our results.

The mean scores for the subscales (6.54 for HADS-A and 5.20 for HADS-D) were similar to the means found in a Brazilian study of nursing professionals working in operating rooms (6.3 and 5.2 for HADS-A and HADS-D respectively)^32^. An international study with different categories of health professionals in hospitals and primary care in Singapore also revealed averages close to our findings (6.9 anxiety and 5.7 depression)^33^.

This study confirms the hypothesis that the HADS is a scale that performs well in screening symptoms of anxiety and depression in PHC health professionals. Although a limited number of studies have addressed this population, there is evidence that the HADS has adequate measurement properties for the sample investigated.

### Limitations and Potential

As in all psychometric studies, the analyses were subject to arbitrary criteria when examining items and factors in the face of variability in the reference values used to interpret the results found in the literature. However, robust methods and commonly accepted cut-off points cited in previous psychometric studies were used.

The sample recruited came from a cross-sectional study involving convenience sampling, so the sensitivity and specificity of the HADS were not measured.

## CONCLUSIONS

The results presented in this psychometric study show that the 14-item HADS instrument with two factors, anxiety and depression, is valid and reliable for screening anxious and depressive symptoms in health professionals working in primary care services in Rio Grande do Sul, Brazil.

Even though a limited number of studies have looked at this population, there is evidence that the HADS has adequate measurement properties for the sample investigated. The importance of applying a valid and reliable instrument is emphasized to ensure the validity of scientific studies and the adequate assessment of mental health status, with a view to applying appropriate interventions for health professionals.

## References

[1] World Health Organization (2017). Depression and other common mental disorders: global health estimates.

[2] Guilland R, Klokner SG, Knapik J, Crocce-Carlotto PA, Ródio-Trevisan KR, Zimath SC (2022). Prevalência de sintomas de depressão e ansiedade em trabalhadores durante a pandemia da Covid-19. Trab Educ Saúde.

[3] Gray P, Senabe S, Naicker N, Kgalamono S, Yassi A, Spiegel JM (2019). Workplace-based organizational interventions promoting mental health and happiness among healthcare workers: a realist review. Int J Environ Res Public Health.

[4] Julio RD, Lourenção LG, Oliveira SM, Farias DHR, Gazetta CE (2022). Prevalência de ansiedade e depressão em trabalhadores da Atenção Primária à Saúde. Cad Bras Ter Ocup.

[5] Søvold LE, Naslund JA, Kousoulis AA, Saxena S, Qoronfleh MW, Grobler C (2021). Prioritizing the mental health and well-being of healthcare workers: an urgent global public health priority. Front Public Health.

[6] Tabur A, Elkefi S, Emhan A, Mengenci C, Bez Y, Asan O (2022). Anxiety, Burnout and depression, psychological well-being as predictor of healthcare professionals' turnover during the covid-19 pandemic: study in a pandemic hospital. Healthcare (Basel).

[7] Enns V, Currie S, Wang J (2015). Professional autonomy and work setting as contributing factors to depression and absenteeism in Canadian nurses. Nurs Outlook.

[8] Baptista MN, Borges L (2016). Revisão integrativa de instrumentos de depressão em crianças/adolescentes e adultos na população Brasileira. Aval Psicol.

[9] DeSousa DA, Moreno AL, Gauer G, Manfro GG, Koller SH (2013). Revisão sistemática de instrumentos para avaliação de ansiedade na população brasileira. Aval Psicol.

[10] Zigmond AS, Snaith RP (1983). The hospital anxiety and depression scale. Acta Psychiatr Scand.

[11] Botega NJ, Bio MR, Zomignani MA, Garcia C, Pereira WA (1995). Transtornos do humor em enfermaria de clínica médica e validação de escala de medida (HAD) de ansiedade e depressão. Rev Saude Publica.

[12] Cassiani-Miranda CA, Scoppetta O, Cabanzo-Arenas DF (2022). Validity of the Hospital Anxiety and Depression Scale (HADS) in primary care patients in Colombia. Gen Hosp Psychiatry.

[13] Piffer L, Schmidt ML, Júnior JM (2021). Ansiedade e depressão entre profissionais de enfermagem em UPA durante a pandemia da covid-19. Rev Psicol Saúde.

[14] Tasnim R, Sujan MS, Islam MS, Ritu AH, Siddique MA, Toma TY (2021). Prevalence and correlates of anxiety and depression in frontline healthcare workers treating people with COVID-19 in Bangladesh. BMC Psychiatry.

[15] Pasquali L (2009). Psicometria Rev Esc Enferm USP.

[16] American Educational Research Association, American Psychological Association, National Council on Measurement in Education (2014). Standards for educational and psychological testing.

[17] Lima DV (2011). Research design: a contribution to the author. Online Braz J Nurs.

[18] Brown TA (2015). Confirmatory factor analysis for applied research.

[19] Hu LT, Bentler PM (1999). Cutoff criteria for fit indexes in covariance structure analysis: conventional criteria versus new alternatives. Struct Equ Modeling.

[20] Khalesi N, Arabloo J, Khosravizadeh O (2014). Psychometric properties of the Persian version of the "Hospital Ethical Climate Survey". J Med Ethics Hist Med.

[21] Hair JF, Black WC, Babin BJ (2009). Análise multivariada de dados.

[22] Kline RB (2005). Principles and practice of structural equation modeling.

[23] Campo-Arias A, Oviedo HC (2008). Propiedades psicométricas de una escala: la consistencia interna. Rev Salud Publica.

[24] Ventura-León JL, Caycho-Rodríguez T (2014). El coeficiente Omega: un método alternativo para la estimación de la confiabilidad. Rev Latinoam Ciencias Sociales.

[25] Pinar R, Celik R, Bahcecik N (2009). Reliability and construct validity of the Health-Promoting Lifestyle Profile II in an adult Turkish population. Nurs Res.

[26] Streiner DL (2003). Starting at the beginning: an introduction to coefficient alpha and internal consistency. J Pers Assess.

[27] Cosco TD, Doyle F, Ward M, McGee H (2012). Latent structure of the Hospital Anxiety And Depression Scale: a 10-year systematic review. J Psychosom Res.

[28] Norton S, Cosco T, Doyle F, Done J, Sacker A (2013). The Hospital Anxiety and Depression Scale: a meta confirmatory factor analysis. J Psychosom Res.

[29] Gough K, Hudson P (2009). Psychometric properties of the Hospital Anxiety and Depression Scale in family caregivers of palliative care patients. J Pain Symptom Manage.

[30] Lloyd M, Sugden N, Thomas M, McGrath A, Skilbeck C (2023). The structure of the Hospital Anxiety and Depression Scale: theoretical and methodological considerations. Br J Psychol.

[31] Stott J, Spector A, Orrell M, Scior K, Sweeney J, Charlesworth G (2017). Limited validity of the Hospital Anxiety and Depression Scale (HADS) in dementia: evidence from a confirmatory factor analysis. Int J Geriatr Psychiatry.

[32] Schmidt DR, Dantas RA, Marziale MH (2011). Ansiedade e depressão entre profissionais de enfermagem que atuam em blocos cirúrgicos. Rev Esc Enferm USP.

[33] Tan BY, Kanneganti A, Lim LJ, Tan M, Chua YX, Tan L (2020). Burnout and associated factors among health care workers in Singapore during the COVID-19 pandemic. J Am Med Dir Assoc.

