# Supplementary material for: Psychometric study of the Hospital Anxiety and Depression Scale with health professionals
Source: Rev Saude Publica. 2025 Jun 11;59:e9. doi: 10.11606/s1518-8787.2025059006420 (PMC12211771; doi:10.11606/s1518-8787.2025059006420)
Supplement: Translation

# Translation: Estudo psicométrico da Hospital Anxiety and Depression Scale com profissionais de saúde

**Authors:** Priscila Pereira da Silva Lopes, Rafaela Schaefer, Juliana Nichterwitz Scherer

**Affiliations:** I Brasil Universidade do Vale do Rio dos Sinos. Programa de Pós-Graduação em Saúde Coletiva. São Leopoldo, RS, Brasil

**Keywords:** Estudo de Validação, Psicometria, Ansiedade, Depressão, Profissionais de Saúde, Atenção Primária

## Abstract

**OBJETIVO:**

Avaliar a validade de estrutura interna e a confiabilidade da versão brasileira da *Hospital Anxiety and Depression Scale* (HADS) para o rastreio de sintomas depressivos e ansiosos em profissionais de saúde da atenção primária.

**MÉTODOS:**

Estudo psicométrico realizado com profissionais de saúde dos serviços da atenção primária à saúde de um município do Rio Grande do Sul, Brasil, em 2023. A qualidade do instrumento HADS foi avaliada pela validade de estrutura interna, por meio da Análise Fatorial Exploratória (AFE), da Análise Fatorial Confirmatória (AFC), e pela consistência interna, pelos coeficientes Alfa de Cronbach e Ômega de McDonald’s.

**RESULTADOS:**

Participaram do estudo 217 profissionais de saúde, de ambos os sexos e de diferentes categorias profissionais, como médicos, enfermeiros, técnicos e auxiliares de enfermagem, dentistas e auxiliares de saúde bucal, e agentes comunitários de saúde. A AFE evidenciou o modelo de dois fatores, ansiedade e depressão, como originalmente proposto. A AFC confirmou que o modelo bidimensional possui bom ajuste para a amostra investigada. Os valores de Alfa e Ômega foram 0,833 e 0,838 para ansiedade; 0,763 e 0,766 para depressão, respectivamente. Ambos indicaram confiabilidade aceitável.

**CONCLUSÕES:**

A HADS é um instrumento válido e confiável para o rastreio de sintomas de ansiedade e depressão em profissionais da atenção primária no Rio Grande do Sul, Brasil.

## INTRODUÇÃO

Ansiedade e depressão são sintomas comuns na população em geral^1^ e na população trabalhadora^2^, incluindo a categoria dos profissionais de saúde^3^. Em um estudo transversal, realizado em 2017, na atenção primária à saúde (APS) de um município de São Paulo, Brasil, a ansiedade e a depressão estiveram presentes em 45,3% e 41,0% dos profissionais, respectivamente^4^.

Os sintomas ansiosos e depressivos acarretam custos individuais e sociais de forma direta e indireta aos profissionais e aos serviços de saúde, por estarem relacionados com: o bem-estar do profissional^5^, a rotatividade profissional^6^, presenteísmo, absenteísmo^7^ e a qualidade do cuidado ofertado^5^. Por esta razão, faz-se importante realizar o rastreamento destes sintomas a fim de identificar condições de saúde e doença e de orientar estratégias de intervenção para promoção e prevenção^5^.

O rastreio dos sintomas de ansiedade e depressão é usualmente feito por meio de instrumentos de medidas, como escalas e questionários. Dentre os diversos instrumentos disponíveis na literatura, está a *Hospital Anxiety and Depression Scale* (HADS)^8,9^. A HADS foi originalmente desenvolvida nos Estados Unidos da América (EUA) para aplicação com pacientes não psiquiátricos em ambiente hospitalar^10^, sendo traduzida e validada para o Brasil em 1995, por Botega et al.^11^. É uma escala autoaplicada, de rápida execução, de fácil compreensão, e sem a necessidade de treinamentos prévios para o seu preenchimento. Tais características fazem deste instrumento um dos mais utilizados para triagem dos sintomas de ansiedade e depressão^12^ com diferentes populações, incluindo os profissionais de saúde^13,14^. Entretanto, muitos destes estudos carecem de avaliação das propriedades psicométricas da HADS para a população e o contexto investigados.

Por meio da psicometria é possível avaliar a qualidade de instrumentos de medida, sendo a validade e confiabilidade as suas principais características^15^. A validade é a propriedade de um instrumento medir exatamente o que se propõe. Dentre os tipos de validade tem-se a validade de estrutura interna, a qual se refere “ao grau em que a evidência e a teoria apoiam as interpretações dos resultados dos testes”^16^. Já a confiabilidade é a capacidade de um instrumento reproduzir resultados precisos. Ambas as propriedades fornecem evidências necessárias sobre a qualidade dos instrumentos e seus resultados, se estes são válidos e confiáveis para a amostra pesquisada^15^.

Considerando que não foi identificado na literatura nenhum estudo psicométrico da HADS aplicado a profissionais de saúde da atenção primária no Brasil, esta pesquisa teve como objetivo determinar a validade de estrutura interna e a confiabilidade da versão brasileira da HADS para o rastreio de ansiedade e depressão em profissionais de saúde da atenção primária de um município da região metropolitana de Porto Alegre, no Rio Grande do Sul.

## MÉTODOS

### Delineamento

Trata-se de um estudo psicométrico do instrumento HADS. Esse processo compreendeu a validação de estrutura interna e a análise da confiabilidade da escala. Esse tipo de estudo contribui para o rigor das pesquisas científicas, por avaliarem os próprios instrumentos de coleta de dados^17^. A psicometria está fundamentada na medida por teoria, isto é, utiliza números e embasamento teórico para assegurar a validade e confiabilidade dos resultados^15^.

### Estudo Principal

Esta pesquisa está vinculada ao estudo transversal: “Contexto Organizacional do Trabalho em Saúde e Saúde do Trabalhador: uma análise do clima ético e fatores associados”, desenvolvido pelo Programa de Pós-Graduação em Saúde Coletiva da Universidade do Vale do Rio dos Sinos (Unisinos) e financiado pela Fundação de Amparo à Pesquisa do Estado do Rio Grande do Sul (Fapergs) pelo edital 10/2021 (ARD/ARC).

### Participantes e Local do Estudo

A população do estudo foi composta por profissionais de saúde que atuavam nos serviços da atenção primária de um município da região metropolitana de Porto Alegre, situado no estado do Rio Grande do Sul, incluindo médicos, enfermeiros, técnicos e auxiliares de enfermagem, dentistas, auxiliares de saúde bucal, agentes comunitários de saúde, fisioterapeutas e psicólogos.

### Procedimentos

A amostra foi selecionada pelo método de conveniência. Os participantes foram recrutados após breve apresentação do estudo às equipes de todas as unidades básicas de saúde (UBS) do município investigado, mediante agendamento prévio, com posterior convite formal para a participação na pesquisa. A amostra final foi composta por 217 profissionais de saúde. Foram incluídos participantes, profissionais de saúde, que atuavam na assistência e excluídos aqueles em cargos exclusivamente administrativos e residentes. A coleta de dado ocorreu presencialmente em abril e maio de 2023, utilizando questionários impressos preenchidos pelos próprios participantes, abordando questões sociodemográficas, a HADS e outras perguntas relacionadas aos demais desfechos presentes no projeto maior.

### Instrumento

A HADS foi desenvolvida por Zigmond e Snaith em 1983^10^. É um instrumento de triagem rápida e autoadministrado, no qual o participante responde com base em como se sentiu durante a última semana. O objetivo da escala é investigar sintomas de ansiedade e depressão, sem a intenção de determinar um diagnóstico clínico. A HADS é composta por duas subescalas, a HADS-A (subescala ansiedade – itens ímpares) e HADS-D (subescala depressão – itens pares) usando uma escala Likert de 0 a 3. Para interpretação dos resultados soma-se a pontuação de cada subescala, variando de 0 a 21 pontos, considerando-se 8 como ponto de corte^10,11^. Utilizou-se neste estudo a versão brasileira da HADS, traduzida e adaptada por Botega et al.^11^.

### Análise Estatística

As análises estatísticas foram realizadas com os *softwares* JASP^®^ versão 0.18.1 e JAMOVI^®^ versão 2.4.1. Foram aplicadas análises descritivas da amostra estimando-se as frequências absolutas e relativas, Análise Fatorial Exploratória (AFE) e Análise Fatorial Confirmatória (AFC) para avaliar a validade de estrutura interna e os coeficientes Alfa de Cronbach (α) e Ômega de McDonald’s (ω) para avaliar a confiabilidade.

Na AFE aplicou-se o método de extração Fatorização do Eixo Principal em combinação com uma rotação Varimax. Para a AFE, teve-se como critérios: o Índice de Kaiser-Meyer-Olkin (KMO) p > 0,05, o Teste de esfericidade de Bartlett p < 0,05 e as cargas fatoriais iguais ou superiores a 0,50^21^.

Na AFC, a estimação dos parâmetros foi realizada pelo método robusto DWLS (*Diagonally Weighted Least Squares*). Os índices da AFC recomendados pela literatura e utilizados neste estudo foram: CFI (*Comparative Fit Index* – índice de ajustamento comparativo), TLI (*Tucker-Lewis Index*), RMSEA (*Root-Mean-Square Error of Approximation* – raiz quadrada da média do erro de aproximação) e SRMR (*Standardized Root Mean Square Residual* – raiz quadrada média residual padronizada)^18,19^. Os valores de referência aceitáveis e esperados para considerar um bom ajuste do modelo foram: CFI e TLI ≥ 0,90 aceitável ou ≥ 0,95 esperado, RMSEA ≤ 0,06 aceitável ou ≤ 0,05 esperado, SRMR ≤ 0,08 aceitável ou ≤ 0,05 esperado^18-20^. Além destes índices, utilizou-se a razão do qui-quadrado (χ^2^) em relação aos graus de liberdade (χ^2^/gl), onde valores de 1 a 3 indicam ajustamento adequado^21,22^.

A confiabilidade da escala foi estimada pelos coeficientes de Alfa de Cronbach e Ômega de McDonald’s. Para ambos os coeficientes se considerou um valor adequado se igual ou superior a 0,70^23,24^, sendo 0,70 aceitável, 0,80 muito aceitável e 0,90 ideal^25^. Valores acima de 0,90 sugerem redundância dos itens^23,26^.

### Aspectos Éticos

Esta pesquisa foi aprovada pelo Comitê de Ética em Pesquisa da Unisinos (n°5.456.590) e pelo Núcleo Municipal de Educação em Saúde Coletiva (Numesc). Manteve-se sigilo das informações coletadas, sem especificação das UBS do município e sem a identificação dos indivíduos. Todos os participantes foram informados sobre os objetivos do estudo e assinaram o termo de consentimento livre e esclarecido.

## RESULTADOS

### Características dos Participantes

As características sociodemográficas e profissionais dos 217 participantes estão apresentadas na Tabela 1. A mediana da idade foi de 40 anos, com predominância de profissionais de saúde do sexo feminino (83,4%), de cor da pele branca (74,1%), casados (53,5%), pertencentes à categoria profissional da enfermagem composta por técnicos e auxiliares (31,3%) e com escolaridade de nível técnico (36,2%). A média de pontuações da HADS-A foi 6,54 (DP ± 4,1) e da HADS-D 5,20 (DP ± 3,34) para toda a amostra.

**Tabela 1:** Características sociodemográficas dos participantes. (n = 217)

Característica	n	%	HADS-A^a^	HADS-D^a^
Idade (Mediana e [IQ])	213	40,0 [33,0; 50,0]	-	-
Sexo				
Feminino	181	83,4	6,71 (4,16)	5,31 (3,34)
Masculino	36	16,6	5,67 (3,75)	4,66 (3,33)
Cor				
Branca	160	74,1	6,37 (4,00)	5,13 (3,32)
Preta	27	12,5	6,59 (4,24)	4,78 (2,97)
Parda	26	12,0	7,46 (4,68)	5,88 (3,92)
Amarela e indígena	3	1,4	7,33 (4,93)	7,00 (3,46)
Estado civil				
Solteiro	100	46,5	6,68 (3,92)	5,29 (3,20)
Casado	115	53,5	6,47 (4,29)	5,13 (3,50)
Profissão				
Médico	34	15,7	6,00 (4,22)	4,71 (3,31)
Enfermeiro	29	13,4	6,34 (3,90)	5,07 (2,79)
Técnico e auxiliar de enfermagem	68	31,3	6,29 (4,10)	4,51 (2,90)
Agente comunitário de saúde	49	22,6	6,98 (4,12)	6,60 (3,76)
Dentista e auxiliar de saúde bucal	33	15,2	6,81 (4,45)	5,03 (3,69)
Outros (fisioterapeuta e psicólogo)	4	1,8	9,00 (1,41)	6,50 (2,08)
Escolaridade				
Fundamental	3	1,4	6,33 (5,13)	6,00 (5,66)
Médio	37	17,4	6,64 (4,01)	5,97 (4,13)
Técnico	77	36,2	6,66 (4,39)	4,87 (3,13)
Graduação	35	16,4	6,20 (3,41)	4,86 (2,86)
Pós-Graduação	61	28,6	6,60 (4,24)	5,38 (3,29)

IQ: intervalo interquartil; HADS-A: subescala ansiedade; HADS-D: subescala depressão

^a^ Média (desvio-padrão).

### Validade de Estrutura Interna da HADS

Os pressupostos da AFE indicaram matriz fatorial apropriada: KMO: 0,91 e Teste de esfericidade de Bartlett < 0,001. Observou-se que a carga fatorial para cada item da HADS variou de 0,53 a 0,72 no fator ansiedade e de 0,44 a 0,63 no fator depressão. Apesar de alguns valores limítrofes, nenhum indicou baixa contribuição à subescala correspondente (Tabela 2).

**Tabela 2:** Carga fatorial dos itens da HADS com profissionais de saúde da atenção primária.

HADS-Item	Carga Fatorial
Subescala Ansiedade	
1. Nervoso ou tenso	0,60
3. Medo de algo horrível	0,72
5. Cheio de preocupação	0,72
7. Tranquilo e relaxado	0,53
9. Medo e aperto no estômago	0,70
11. Inquieto	0,58
13. Pânico	0,67
Subescala Depressão	
2. Gosta das mesmas coisas	0,44
4. Risada e diversão	0,57
6. Sente-se alegre	0,63
8. Lentidão	0,54
10. Cuidar da aparência	0,57
12. Esperança para o futuro	0,62
14. Prazer em livros, TV ou rádio	0,53

HADS: *Hospital Anxiety and Depression Scale*

A AFC da HADS total e por subescalas apresentou um bom modelo de ajuste para a amostra em estudo, conforme valores apresentados na Tabela 3.

**Tabela 3:** Análise fatorial confirmatória da HADS.

Índice	Valor esperado	Valor aceitável	HADS-total	Conclusão HADS-total	HADS-A	Conclusão HADS-A	HADS-D	Conclusão HADS-D
χ^2^ (gl)	-	-	129 (76)	-	18,500 (14)	-	23.6 (14)	-
p valor χ^2^	> 0,05	-	< 0,001	Mal ajuste	0,185	Bom ajuste	0,051	Bom ajuste
χ^2^/gl	-	1–3	1,697	Adequado	1,320	Adequado	1,68	Adequado
CFI	≥ 0,95	0,90	0,940	Aceitável	0,990	Esperado	0,963	Esperado
TLI	≥ 0,95	0,90	0,929	Aceitável	0,985	Esperado	0,945	Aceitável
RMSEA	≤ 0,50	0,60	0,056	Aceitável	0,038	Esperado	0,056	Aceitável
IC90% RMSEA	-	-	0,039–0,073	-	0,000–0,080	-	0,000–0,094	-
SRMR	≤ 0,50	0,80	0,050	Esperado	0,028	Esperado	0,038	Esperado

χ^2^: teste qui-quadrado; gl: graus de liberdade; CFI: índice de ajustamento comparativo; TLI: índice de Tucker Lewis; RMSEA: raiz quadrada da média do erro de aproximação; IC: intervalo de confiança; SRMR: raiz quadrada média residual padronizada

Ans: ansiedade; Dep: depressão; HADS1: *Hospital Anxiety and Depression Scale item number* 1

A Figura apresenta o modelo estrutural da versão brasileira da HADS com profissionais de saúde da atenção primária e mostra que ambas subescalas (ansiedade e depressão) estão positivamente correlacionadas uma com a outra.

**Figura:** Diagrama fatorial da HADS para a amostra em estudo.

### Confiabilidade da HADS

O coeficiente alfa encontrado foi de 0,879 para HADS-total, 0,833 para HADS-A e 0,763 para HADS-D. Adicionalmente, os valores de ômega foram 0,881 para HADS-total, 0,838 para HADS-A e 0,766 para HADS-D. Observa-se que os valores mostram consistência interna aceitável e muito aceitável (0,70–0,80) não havendo redundância na escala, visto que nenhum valor foi superior a 0,90.

A mudança no valor da consistência interna, avaliada pelos coeficientes alfa e ômega, foi testada conforme a retirada de itens da escala para avaliar se algum item se apresentava destoante com a escala e/ou seu fator, analisando se a escala/subescala ficaria melhor sem ele. Os resultados apresentam que todos os itens são necessários para a composição da escala. Ademais, a correlação entre o item e a pontuação total da escala, apresentada como correlação-item total, apresenta adequada correlação, exceto para os itens 2, 4 e 14 (Tabela 4).

**Tabela 4:** Características de confiabilidade da HADS.

Item	Média (DP)	Correlação item-total	Se o item for eliminado	
			α de Cronbach	ω de McDonald’s
HADS-1	1,23 (0,76)	0,528	0,872	0,874
HADS-2	0,80 (0,67)	0,401	0,878	0,879
HADS-3	0,89 (0,95)	0,626	0,867	0,869
HADS-4	0,32 (0,61)	0,485	0,874	0,875
HADS-5	1,44 (0,90)	0,637	0,867	0,868
HADS-6	0,89 (0,72)	0,535	0,872	0,873
HADS-7	1,03 (0,84)	0,572	0,870	0,871
HADS-8	1,15 (0,81)	0,517	0,873	0,874
HADS-9	0,71 (0,72)	0,608	0,869	0,870
HADS-10	0,72 (0,80)	0,523	0,873	0,874
HADS-11	0,77 (0,89)	0,554	0,871	0,872
HADS-12	0,77 (0,83)	0,593	0,869	0,870
HADS-13	0,44 (0,67)	0,615	0,869	0,869
HADS-14	0,56 (0,73)	0,473	0,875	0,876

DP: desvio-padrão

## DISCUSSÃO

O presente estudo avaliou as propriedades psicométricas da HADS, validade de estrutura interna e confiabilidade, em uma amostra de profissionais de Saúde da APS de um município do Rio Grande do Sul, Brasil. Os resultados da AFE indicaram que a matriz fatorial da HADS é apropriada para esta população. A estrutura de dois fatores – ansiedade e depressão – apresentou ajuste satisfatório na AFC e mostrou confiabilidade aceitável para a escala total e subescalas pelo Alfa de Cronbach e Ômega de McDonald’s. Ademais, todos os itens estiveram correlacionados com seus respectivos fatores.

Um debate muito importante sobre a HADS é sobre a sua dimensionalidade. Alguns autores apoiam a estrutura unidimensional da escala, por acreditarem que ela não diferencia claramente os construtos de ansiedade e depressão e, assim, propõem que seja usada como uma medida unidimensional geral de sofrimento emocional^27^. Outros autores apontam que um modelo de dois fatores se ajusta melhor à HADS, quando comparado com um modelo de um (unifatorial) ou de três fatores^28^. Gough e Hudon^29^ exploraram modelos de dois e três para uma amostra australiana de cuidadores de pacientes com câncer e descobriram que o melhor modelo foi o bidimensional. A estrutura de dois fatores foi encontrada na maioria dos estudos de validade, mesmo que com amostras diferentes, apresentando índices de bom ajuste e fornecendo suporte para a estrutura bidimensional original da HADS^27,28,30^, corroborando com os achados do nosso estudo.

A falha na diferenciação dos constructos não inviabiliza os resultados da HADS^28^, contudo, recomenda-se que seja usada como medida separada para ansiedade e depressão^12^. Comentários em direção semelhante foram feitos em um estudo com pessoas que sofrem de demência, o qual mostrou que a HADS se apresentou adequada para ser usada em seu formato original, reforçando a recomendação de que os fatores ansiedade e depressão devem ser medidos e analisados separadamente^31^.

Em relação aos índices fatoriais da HADS, até o momento, há apenas um estudo semelhante a este, realizado com uma amostra de profissionais de saúde a partir de um estudo transversal. Apesar de apresentar índices e valores semelhantes e utilizar o mesmo referencial de base para os valores de referência na AFC, o estudo não foi realizado no contexto da APS e contou com uma amostra de 803 profissionais de saúde de Bangladesh^14^.

Neste estudo, os índices da AFC apresentaram um bom modelo de ajuste, confirmando a estrutura teórica da HADS. A análise das cargas fatoriais revelou que os itens da HADS se agruparam de modo claro em seus respectivos fatores de ansiedade e depressão, corroborando com a estrutura bidimensional proposta no modelo original de Zigmond e Snaith^10^. Apenas o item 2, pertencente ao fator depressão, apresentou peso fatorial inferior a 0,50 (0,44).

Os indicadores da consistência interna (α) da HADS apresentaram-se satisfatórios quando comparados aos valores recomendados pela literatura^23-25^ e a estudos prévios^13,14^. No entanto, não foi encontrada nenhuma pesquisa que aplicou o ω de McDonald’s com profissionais de saúde para avaliar a confiabilidade, fato que dificulta a comparação dos nossos resultados.

As médias das pontuações das subescalas (6,54 para HADS-A e 5,20 para HADS-D) foram semelhantes às médias encontradas em um estudo brasileiro com profissionais de enfermagem que atuam em blocos cirúrgicos (6,3 e 5,2 para HADS-A e HADS-D respectivamente)^32^. Um estudo internacional, com diversas categorias de profissionais de saúde de hospitais e da atenção primária em Cingapura, também revela médias aproximadas aos nossos achados (6,9 ansiedade e 5,7 depressão)^33^.

O presente estudo confirma a hipótese de que a HADS é uma escala que apresenta bom desempenho na triagem dos sintomas de ansiedade e depressão em profissionais de saúde da APS. Embora um número limitado de estudos tenha abordado esta população, há evidências de que a HADS tem propriedades de medida adequadas à amostra investigada.

### Limitações e Potencialidades

Como em todos os estudos psicométricos, as análises estavam sujeitas a critérios arbitrários ao examinar itens e fatores diante variabilidade de valores de referência de base para a interpretação dos resultados encontrados na literatura. Contudo, utilizaram-se métodos robustos e pontos de corte comumente aceitos e citados em estudos psicométricos anteriores.

A amostra recrutada foi proveniente de um estudo transversal envolvendo amostragem por conveniência, de modo que não foi realizada sensibilidade e especificidade da HADS.

## CONCLUSÕES

Os resultados apresentados neste estudo psicométrico mostram que o instrumento HADS de 14 itens e com dois fatores, ansiedade e depressão, é válido e confiável para triagem dos sintomas ansiosos e depressivos em profissionais de saúde que atuam nos serviços da atenção primária no Rio Grande do Sul, Brasil.

Mesmo com um número limitado de estudos que tenha abordado esta população, há evidências de que a HADS tem propriedades de medida adequadas à amostra investigada. Ressalta-se a importância de aplicar um instrumento válido e confiável a fim de assegurar a validade dos estudos científicos e a avaliação adequada do estado de saúde mental, visando a aplicação de intervenções apropriadas para profissionais de saúde.
